# Performance of Intraoperative Contrast-Enhanced Ultrasound (Io-CEUS) in the Diagnosis of Primary Lung Cancer

**DOI:** 10.3390/diagnostics14151597

**Published:** 2024-07-24

**Authors:** Martin Ignaz Schauer, Ernst Michael Jung, Hans-Stefan Hofmann, Natascha Platz Batista da Silva, Michael Akers, Michael Ried

**Affiliations:** 1 Department of Thoracic Surgery, University Medical Center Regensburg, Franz-Josef-Strauss-Allee 11, 93053 Regensburg, Germany; hans-stefan.hofmann@ukr.de (H.-S.H.); michael.ried@ukr.de (M.R.); 2 Institute for Radiology, University Medical Center Regensburg, Franz-Josef-Strauss-Allee 11, 93053 Regensburg, Germany; ernst-michael.jung@ukr.de (E.M.J.); natscha.platz-batista-da-silva@ukr.de (N.P.B.d.S.); michael.akers@ukr.de (M.A.)

**Keywords:** CEUS, intraoperative CEUS, CEUS lung cancer

## Abstract

Background: Suspicious tumors of the lung require specific staging, intraoperative detection, and histological confirmation. We performed an intrathoracic, intraoperative contrast-enhanced ultrasound (Io-CEUS) for characterization of lung cancer. Methods: Retrospective analysis of prospectively collected data on the application of Io-CEUS in thoracic surgery for patients with operable lung cancer. Analysis of the preoperative chest CT scan and FDG-PET/CT findings regarding criteria of malignancy. Immediately before lung resection, the intrathoracic Io-CEUS was performed with a contrast-enabled T-probe (6–9 MHz—L3-9i-D) on a high-performance ultrasound machine (Loqic E9, GE). In addition to intraoperative B-mode, color-coded Doppler sonography (CCDS), or power Doppler (macrovascularization) of the lung tumor, contrast enhancement (Io-CEUS) was used after venous application of 2.4–5 mL sulfur hexafluoride (SonoVue, Bracco, Italy) for dynamic recording of microvascularization. The primary endpoint was the characterization of operable lung cancer with Io-CEUS. Secondly, the results of Io-CEUS were compared with the preoperative staging. Results: The study included 18 patients with operable lung cancer, who received Io-CEUS during minimally invasive thoracic surgery immediately prior to lung resection. In the chest CT scan, the mean size of the lung tumors was 2.54 cm (extension of 0.7–4.5 cm). The mean SUV in the FDG-PET/CT was 7.6 (1.2–16.9). All lung cancers were detected using B-mode and power Doppler confirmed macrovascularization (100%) of the tumors. In addition, Io-CEUS showed an early wash-in with marginal and mostly simultaneous central contrast enhancement. Conclusions: The intrathoracic application of Io-CEUS demonstrated a peripheral and simultaneous central contrast enhancement in the early phase, which seems to be characteristic of lung cancer. In comparison to preoperative imaging, Io-CEUS was on par with the detection of malignancy and offers an additional tool for the intraoperative assessment of lung cancer before resection.

## 1. Introduction

According to the current WFUMB-EFSUMB “Guidelines and Good Clinical Practice Recommendations for Contrast Enhanced Ultrasound in the Liver” and “on non-hepatic applications”, contrast-enhanced ultrasound (CEUS) can be used in a variety of diagnostic and therapeutic settings [1]. Both percutaneous and intraoperative applications are recommended, with percutaneous applications usually requiring smaller amounts of contrast agent [2,3,4]. The intravenous application of a gas-containing, micrometer-sized contrast agent in the form of microbubbles, which are put into a state of persistent oscillation by suitable ultrasound applications, can increase the echogenicity of the blood and thus enable the sonographic visualization of blood vessels and the perfusion status of organs [5,6]. For example, several groups of authors have demonstrated the high value of CEUS in the diagnosis of thyroid tumors [7,8,9,10]. They showed that contrast enhancement patterns were significantly different in benign and malignant lesions. Platz Batista da Silva N. et al. demonstrated that in a study involving 54 focal pancreatic lesions (FPLs), intraoperative CEUS showed a sensitivity of 100% and a specificity of 40% in distinguishing malignant from benign lesions, with an accuracy of 83.3%. Shear wave elastography (SWE) showed a sensitivity of 74.4% and a specificity of 46.7% using specific cutoff values. Combining both techniques resulted in an accuracy rate of 76%, with immediate surgical impact observed in 29.6% of cases [11]. With comparable and proportionally higher sensitivity and specificity rates of CEUS compared to computed tomography (CT) in the detection and characterization of hepatic malignancies, several studies have already confirmed the high diagnostic value of Io-CEUS, particularly in liver tumor diagnostics [12,13,14,15,16]. Werner J. et al. demonstrated that intraoperative ultrasound (IOUS) with CEUS and elastography accurately identified 42 malignant tumors and four benign lesions, yielding a sensitivity of 97.7%, specificity of 57.1%, positive predictive value of 93.3%, and negative predictive value of 80%. Surgeons’ specific questions were successfully answered in 98% of cases, and in 76% of cases, IOUS led to modifications (42%) or fundamental changes (34%) in the planned surgical approach, significantly impacting therapy outcomes. Despite only a slight difference in procedural times between setup and return transport, the average examination time of 14 min constituted only one-third of the overall time requirement [17]. The use of CEUS also offers clear advantages in the intraoperative setting. Improved detection of liver lesions has been confirmed, and surgical performance has been increased [3,4,18,19,20,21].

To date, there have been a few studies on transthoracic CEUS. New potential applications were described, for example, by the authors Jung et al. who investigated the applicability of CEUS in intensive care units for severe cases of COVID-19 infection during the COVID pandemic [22]. There were indications of a possible benefit of therapy monitoring. Some studies have described CEUS-assisted biopsy as an effective and safe method for pleural lesions with increased diagnostic accuracy [23,24,25,26]. In a prospective study involving 460 patients, Sun W. et al. found that CEUS demonstrated higher rates of internal necrosis and peripheral vessel visualization compared to conventional ultrasound imaging. CEUS-guided biopsy sampling exhibited a diagnostic accuracy of 98.91%, with a microbiological diagnostic yield of 71.88% in infectious lesions. In cases of combined pleural effusion, CEUS-guided biopsy significantly increased the diagnostic yield, particularly in malignant and infectious lesions, with no reported serious adverse events [23]. Our research group was the first to perform Io-CEUS in thoracic surgery and was able to demonstrate its feasibility and the possibility of characterizing lung tumors immediately prior to lung resection [27]. In the context of lung cancer screening, diagnostic tools are becoming increasingly important in order to avoid initiating unnecessary or even incorrect therapies that could ultimately harm patients [28,29]. In addition, due to the continuing high incidence of lung cancer and increasingly upcoming screening examinations, a further increase in solitary pulmonary nodules (SPNs) of unclear entity, which must be histologically confirmed, is to be expected [30]. Consequently, in addition to preoperative staging, we also need innovative procedures to find SPNs intraoperatively on the non-ventilated, deflated lung and, at best, to characterize them already with regard to their entity. Until now, the extent of resection in cases of SPNs of unclear dignity is decisive and continues to depend on the intraoperative frozen section result.

This retrospective comparative study describes the sonographic characteristics of operable primary lung cancer in intraoperative contrast-enhanced ultrasound (Io-CEUS) in minimally invasive thoracic surgery. In addition, results on the characterization of lung cancer in comparison with preoperative staging are described on the basis of their malignancy criteria.

## 2. Materials and Methods

### Study Design

This was a retrospective analysis of data (subgroup) from a prospective, observational, single-center study on patients, who underwent minimally invasive thoracic surgery (video-assisted thoracic surgery = VATS) for operable lung cancer and who received intrathoracic Io-CEUS immediately before lung resection. All patients were informed preoperatively about the additional Io-CEUS and gave written informed consent. The study received a positive ethics vote from the local ethics committee (reference: 21-2301-101). For the Io-CEUS procedure, we refer to our previous work [27]. The ultrasound examination was performed by an experienced radiologist with DEGUM Level III certification. The probe was operated by the surgeon. Through an auxiliary incision of approximately 4 cm, a T-probe (6–9 MHz, L3-9i-D, GE) was introduced into the thorax. The tumor was visualized in B-mode using a high-performance ultrasound machine (LOGIQ E9/10). Power Doppler and color-coded Doppler sonography (CCDS) were used to depict the macrovascularization, and contrast-enhanced ultrasound was utilized for the microvascularization. From the time of contrast application, a timer was started and a cine loop of 60 s was recorded. The ultrasound probe captures a series of 2D images over a specific area during the examination. These stored 2D images are processed in a dedicated processor within the ultrasound machine. This process involves analyzing the positions of structures and the distribution of the contrast agent. The ultrasound machine uses the processed data to generate a 3D image that represents the spatial arrangement of structures and the distribution of the contrast agent.

Only patients with primary and operable lung cancer were included. Preoperative imaging using contrast-enhanced CT-scan of the chest and FDG-PET/CT was used for staging the suspicious malignant lung tumor. The CT examinations were analyzed by radiologists, which included the tumor size, shape, and localization of the lung tumor. The FDG-PET/CT images were analyzed by nuclear medicine specialists, and the standard uptake values (SUV) were determined. These were categorized into three categories based on their SUV level: mild (SUV ≤ 2.5), moderate (SUV > 2.5 and ≤10), and intense (SUV > 10). In addition to the criteria mentioned above, age, smoking history, and previous tumor history were used to calculate the probability of malignancy according to the Herder model as recommended in the German guidelines for lung cancer [31].

## 3. Results

### 3.1. Patient Characteristics

A total of 18 patients (female *n* = 9) with a mean age of 64.3 ± 7.3 years were included. The smoking status was positive in 14 patients. Six patients had a history of malignancy within the last five years. The preoperative probability of malignancy according to the Herder model was 14.3–97.3% (mean 82%). In the imaging, all pulmonary findings were classified as malignant and worthy of clarification. The resection was sonography-guided with marking of the tumor area in all cases without complications. There was no contrast intolerance reaction noted during Io-CEUS. In the histological work-up, the mean size was 2.72 cm ± 1.04 cm (max 0.6 to 4.5 cm). Histology confirmed primary lung cancer in all patients with the subtypes of adenocarcinoma (*n* = 10), squamous cell carcinoma (*n* = 3), and carcinoid (*n* = 5).

### 3.2. Preoperative Imaging

All data regarding the three different diagnostic tools are shown in Table 1. The chest CT scan showed that the lung tumors had an extension of 0.7–4.5 cm (mean 2.53 cm) and a distance to the lung surface (visceral pleura) of 0.0–4.6 cm (mean 1.81 cm). Six lesions had a spiculated shape, whereas eight were sharply defined, three were blurred, and one tumor had streaky extensions. Based on the size, 16 tumors (89%) were classified as malignant. In combination with the shape, all 18 tumors (100%) were preoperatively classified as malignant. No preoperative differentiation could be made between primary lung cancer and metastasis. The tumors were located in the upper lobe (*n* = 11), in the lower lobe (*n* = 6), and in the middle lobe (*n* = 1). The SUV was determined 1.2–16.9 (mean 7.6). In one case, no FDG-PET/CT was performed. In a further case, no SUV was documented but strong contrast agent uptake was described. Six patients had a strong (SUV > 10), eight patients a moderate (SUV > 2.5 and ≤10), and two patients had only low (SUV ≤ 2.5) nucleotide uptake.

### 3.3. B-Mode and Power Doppler/CCDS

In all cases, localization of the tumor was possible by B-mode and could be visualized by power Doppler or CCDS. Similarly, B-mode was used to visualize and describe the shape as well as echogenicity of the tumor. Here, primary lung cancer had jagged margins in B-mode and was predominantly inhomogeneous in echogenicity. Evidence of necrotic areas was present in some tumors, especially in larger tumors (>3 cm). CCDS or power Doppler was able to visualize the macrovascularization behavior of the tumors. Primary lung cancer showed a centrally located vascular gating.

### 3.4. Io-CEUS

All tumors already showed central and contemporaneous peripheral contrast enhancement at t = 9–12 s, which extended over the entire tumor as time progressed. Approximately 83% (15/18) of lung tumors showed at t = 9–13 s a peripheral and simultaneous central contrast enhancement, which extended over the entire tumor as time progressed. Only a peripheral enhancement was documented in two patients; only a central enhancement was seen in one patient.

### 3.5. Case 1

**Figure 1:**
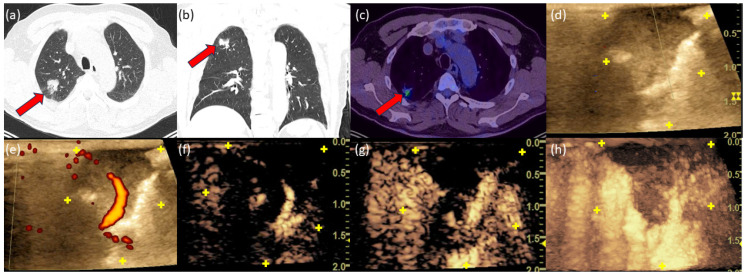
Chest CT scan in axial view (**a**) and coronal view (**b**) showing a suspicious tumor in the upper lobe (red arrows). FDG-PET/CT revealed an SUV of 4.8 (**c**). In B-mode, the dimensions of the tumor can be seen (**d**). The yellow “+” marks the tumor margin. (**e**) The power Doppler with central and peripheral vessel sections. In CEUS (**f**) at t = 9 s, the central and peripheral contrast agent uptake can be seen, which increasingly spreads throughout the tumor over time (**g**) at t = 14 s. (**h**) The 3D reconstruction of the CEUS confirmed a marginal and central contrast center uptake.

A 65-year-old male patient with a positive smoking history and no history of extrathoracic tumor. The chest CT scan showed a 1.6 cm SPN in the right upper lobe, which is partially indistinct. In FDG-PET/CT, there was a moderate FDG uptake. The calculation of the probability of malignancy according to Herder was 75.5%. Io-CEUS showed a peripheral and central enhancement in the early phase of contrast agent uptake (Figure 1). Histological examination confirmed primary pulmonary adenocarcinoma.

### 3.6. Case 2

**Figure 2:**
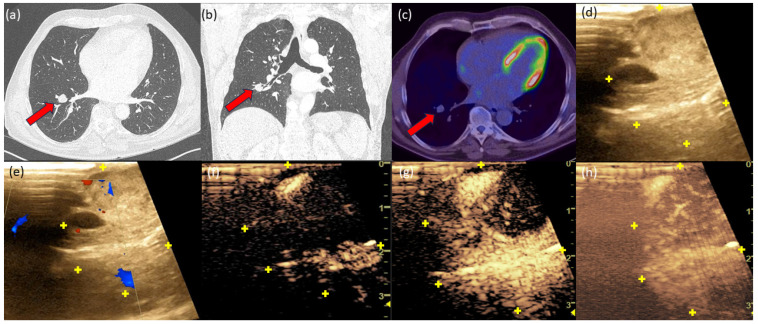
Chest CT-scan axial (**a**) and coronary (**b**) showed a clearly defined SPN with a diameter of 1.6 cm in the right lower lobe with an SUV of 13.7 in the FDG-PET/CT (**c**). The red arrow marks the tumor. In B-mode (**d**), we see a partially sharply demarcated, echo-heterogeneous tumor. The yellow “+” marks the tumor margin. In CCDS (**e**), a peripheral vessel section is distinguishable apically at the tumor margin and centrally. In the early contrast agent phase (**f**) at t = 12 s, a clear central and peripheral contrast can be seen, which rapidly spreads over the tumor (**g**) at t = 18 s. (**h**) The 3D reconstruction of the tumor.

A 64-year-old patient with a positive smoking history and a suspicious pulmonary tumor in the right lower lobe (Figure 2). There was a history of papillary bladder carcinoma. A malignancy probability of 85% was calculated according to the Herder model. Io-CEUS showed an early (t = 12 s) central and simultaneous peripheral contrast enhancement, which extended over the entire tumor over time. The intraoperative frozen section revealed a typical carcinoid of the lung.

### 3.7. Case 3

**Figure 3:**
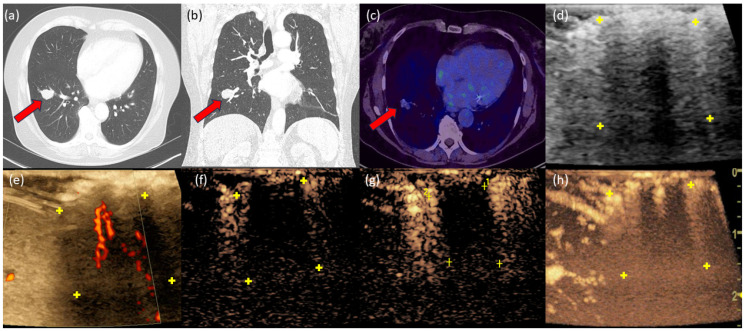
In the CT thorax, a clearly partially lobulated SPN can be delineated in the right lower lung lobe (**a**,**b**). The red arrow marks the tumor. In the PET-CT (**c**), the SPN appears faintly. In B-mode (**d**), a partially hypo- and partially hyperechoic structure can be delineated. (**e**). The yellow “+” marks the tumor margin. The power Doppler with peripheral and central vessel sections. In CEUS, an early peripheral contrast enhancement is observed in the wash-in phase at t = 12 s (**f**), which intensifies over time at t = 19 s (**g**). The 3D reconstruction shows central contrast agent uptake with a large void (**h**).

A 62-year-old patient with a cumulative history of 30 pack-years and a history of endometrial carcinoma presented with a 2.7 cm SPN in the right lower lobe with an SUV max of 2.0 (Figure 3). The Herder model indicated a malignancy probability of 60%. Power Doppler demonstrated peripheral macrovessel vascularization. In CEUS, early peripheral contrast enhancement was observed. As time progressed, the enhancement extended from the peripheral region to a central section of the tumor. Histopathologically, this was identified as a 2.8 cm typical carcinoid embedded in fibrotic stroma.

## 4. Discussion

Our first clinical results and practical experience confirmed that operable lung cancer can be detected by Io-CEUS, but above all, they can be characterized more specifically by the additional use of color-coded Doppler sonography (CCDS) or power Doppler and the application of contrast medium (CEUS). The insertion of the T-probe into the chest via minimally invasive access of about 4 cm length is technically possible. However, we must note that the intraoperative and intrathoracic handling of the T-probe via such a small approach is technically difficult, so an endoscopic ultrasound probe with the necessary functions should be used here in the future. However, it would then also be a prerequisite that this endoscopic probe also has other functions, which are absolutely necessary for the specific description of the tumor. We have already been able to test an endoscopic probe [32]. The surgical handling was significantly simplified. Further studies will follow in the future.

Other studies have already investigated the significance of intrathoracic ultrasound with regard to the possibility of detecting pulmonary nodules and compared the results with intraoperative bimanual palpation [33,34,35,36]. Here, the studies confirmed that intrathoracic ultrasound is a suitable tool compared to conventional palpation. In particular, Khereba et al. demonstrated the high utility of intraoperative ultrasound for pulmonary nodule detection also in minimally invasive thoracic surgery [35]. This reduced the rate of conversions from VATS to conventional open thoracotomy with limited manual palpation capability in the minimally invasive approach. Increasingly, robot-assisted procedures are being adopted in thoracic surgery [37,38]. Currently, the lack of palpable control when using this robotic system is a major disadvantage, as surgeons are unable to locate lesions with their fingers or a device as is possible with VATS.

In our study, we went one step further and focused instead on the characterization of pulmonary tumors using CCDS to visualize macrovascularization and Io-CEUS to visualize microvascularization [27]. Io-CEUS is already used as standard in liver tumor surgery to differentiate between malignant and benign lesions and metastases and has found its way into international ultrasound guidelines [39,40]. The resulting advantages of the examination method directly prior to surgical resection have a significant influence on the surgical procedure [16,17,41].

Our data provide evidence of different vascularization and contrast agent kinetics depending on the tumor entity. Primary lung cancers have in common a peripheral and usually at the same time centrally beginning contrast agent enhancement in the early phase after contrast agent application. In one tumor, only an early peripheral enhancement without central enhancement was detected. In this case, however, there was a large central necrosis zone. In two other cases, the exact determination of the contrast agent enhancement was difficult due to the size of only 7 mm. There were also differences in further enhancement. One tumor was limited to central and peripheral enhancement over time. In contrast, another tumor showed ubiquitous contrast agent enhancement over time. In the B-mode, the tumors differed in size but also in a centrally hyperechogenic structure, compatible with possible tumor necrosis, which could have influenced the expansion of the contrast agent.

Differentiation between malignant and benign SPN is possible on the basis of morphological criteria such as density, size, and delimitation using CT imaging alone. However, further classification into primary lung cancer and lung metastases has not yet been successful in many cases. Ultimately, biopsies or follow-up examinations are required to confirm the diagnosis [42]. In combination with FDG-PET/CT, higher sensitivity and specificity rates in the differentiation between malignant and benign SPNs have been achieved in the literature.

Kim et al., for example, compared the performances of FDG-PET alone, CT alone, and FDG-PET/CT [43]. They reported a sensitivity and specificity of 93% and 31% for CT, 69% and 85% for PET, and 97% and 85% for PET/CT, respectively. Several other studies underlined the value of joint morphological and functional analysis [44,45]. In these studies, the interpretation of the hypermetabolic foci seen on PET images also took into account the nodule’s morphological features (regular, lobulated, or spiculated margins; presence of central, peripheral, or popcorn calcifications; etc.), its density (strong attenuation, ground glass opacity, fluid, necrosis), and lesion enhancement in the event of injection of iodinated contrast agent. The subjective categorization of SUV values in FDG-PET/CT also shows weaknesses here [46]. A meta-analysis of FDG-PET and PET/CT studies for the initial diagnosis of lung masses or known lung cancer evaluated the utility of SUV for prognostic stratification of non-small cell lung cancer. These studies employed diverse calculation methods and thresholds for SUV values, resulting in variable outcomes. Nevertheless, most studies demonstrated significant differences in overall survival based on SUV values, with higher values associated with an increased risk of premature death. The combined hazard ratio for patients with high SUV values was 2.27, indicating a markedly elevated mortality risk [47,48,49]. However, due to the risk of false-negative findings, PET is not indicated for characterizing small nodules. The American College of Chest Physicians recommended an FDG-PET/CT scan for SPNs > 8 mm in diameter [50]. This threshold of 8 mm was set to take into account the spatial resolution of PET systems, due to the significant risk of false-negative findings for small lesions. The main cancers for which false-negative findings are observed are typical carcinoid tumors and certain early forms of adenocarcinoma, such as adenocarcinoma in situ [51].

A validated calculation model (e.g., Herder Model, Mayo Clinic, Brock Model) can be used to estimate the probability of malignancy of a newly occurring SPN [29,31]. However, only statements on the probability of malignancy can be made here, but none on the differentiation between primary lung cancer and lung metastasis.

To date, it should be emphasized that it appears possible to differentiate between primary lung cancer and metastasis using intrathoracic Io-CEUS. Analogous to the use of Io-CEUS in liver surgery, the contrast agent kinetics of primary lung cancer can be explained by tumor angiogenesis [52]. However, the blood supply to the liver differs from that to the lungs. Here, an arterial, a portal venous, and a late venous phase can be distinguished. Based on wash-in and wash-out kinetics, the liver lesion can be characterized as an arterial hypervascularized or irregularly vascularized lesion with wash-out in the portal venous to late phase being classified as malignant. Increasing wash-in to portal venous and late phase is considered a criterion of benignity [1,53]. The lung, on the other hand, presents an entirely different perfusion situation with the vasa privata et publica. Here, contrast enhancement depends on the perfusion of the tumor by the lung’s own vessels or by the pulmonary arteries. From which vascular system the tumor is fed is currently unclear [54]. A wash-out has been observed only in isolated cases. A possible hypothesis would be that an early and rapid contrast enhancement, for example, from t = 6 s, is observed in lung tumors, which has a direct connection to the pulmonary arterial supply, whereas a later enhancement, t = 15 s, is rather supplied by the bronchial arteries, analogous to the CT contrast-enhanced protocol commonly used in clinical practice. Tumor perfusion turns out to be complex and multimodal. This should be explored in further studies. In all cases, CCDS was feasible to visualize macrovascularization and Io-CEUS to visualize microvascularization. Quantification using TIC analysis is possible under optimal conditions. Differentiation of tumor entities based on the contrast agent behavior thus appears possible. TIC analyses and collection of parametric data could confirm the assumptions in the future. However, the examination method is highly dependent on the experience of the surgeon and the ultrasound examiner. A clean contact surface with good acoustic conditions is necessary for meaningful image quality. Further differentiation using elastography (sharewave analysis) would be desirable in the future.

## 5. Limitations

As a limitation of the study, the small sample size must be mentioned. Due to the description of data obtained from a small number of cases, meaningful statistical analysis is not feasible. Additionally, the retrospective study design represents another limitation.

## 6. Conclusions

Io-CEUS using a T-probe and high-performance ultrasound device can be used in minimally invasive thoracic surgery (VATS) to detect and characterize operable lung cancer. This innovative technique could be a new method for intraoperative visualization of pulmonary nodules or tumors. Particularly in VATS, Io-CEUS offers entirely new possibilities for intraoperative imaging. This is because pulmonary nodules can often not be visualized or palpated during VATS. Io-CEUS has advantages in detecting pulmonary nodules, visualizing pulmonary lesions, and characterizing them through perfusion and contrast agent uptake. The differentiation of individual tumor entities directly prior to surgical resection appears to be possible with the aid of high-performance sonography including power Doppler or CCDS and contrast agent application. In comparison to preoperative imaging, Io-CEUS was on par with the detection of malignancy. Further examinations and analyses are carried out by our working group.

## Figures and Tables

**Table 1:** Study sample including preoperative imaging, risk calculation, and intraoperative characterization using Io-CEUS.

Patients	Chest CT Scan		FDG-PET/CT	Herder Model	CCDS	Io-CEUS	Histology
	Size (cm)	Configuration	SUV Max	Probability of Malignancy (%)	Vascularization	Contrast Enhancement	Differentiation of Lung Cancer
1	3.4	Soft tissue dense mass	13	95	Central	Central and peripheral	Adenocarcinoma
2	0.7	Spiculated	5	76.4	Central	Central	Adenocarcinoma
3	0.7	Clearly definable	1.2	14.3	None	Peripheral	Carcinoid
4	3.6	Irregularly limited	6.3	94	Central	Central and peripheral	Adenocarcinoma
5	3.2	Soft tissue dense mass	11	91.7	Central	Central and peripheral	Adenocarcinoma
6	1.9	Irregularly limited	3.8	71.7	Central and peripheral	Central and peripheral	Adenocarcinoma
7	2.7	Lobulated	2	60	Peripheral	Central and peripheral	Carcinoid
8	1.6	Clearly definable	13.7	85	Central	Central and peripheral	Carcinoid
9	2.6	Spiculated	16.9	92.4	Peripheral	Peripheral and sporadic central	Adenocarcinoma
10	1.6	Irregularly limited	4.8	75.5	Central and peripheral	Central and peripheral	Adenocarcinoma
11	3.8	Clearly definable	5.8	86.5	Central and peripheral	Central and peripheral, necrotic	Carcinoid
12	4.5	Spiculated	4.2	97.1	Peripheral	Peripheral, central necrotic	Squamous cell carcinoma
13	3.8	Spiculated	Intense	95.4	Peripheral	Peripheral and central	Adenocarcinoma
14	2.5	Cavernous	-	73	Central and peripheral	Peripheral and central	Squamous cell carcinoma
15	3.8	Lobulated	11.7	97.3	Central and peripheral	Peripheral and central	Adenocarcinoma
16	1.5	Spiculated	4.0	80	Central and peripheral	Peripheral and central	Adenocarcinoma
17	1.4	Spiculated	16.1	95.4	Central and peripheral	Central and peripheral	Squamous cell carcinoma
18	2.4	Clearly definable	3.1	91.5	Central and peripheral	Central and peripheral	Carcinoid

## Data Availability

Data are available on request due to privacy and ethical restrictions.

## Abbreviations

Io-CEUS	intraoperative contrast-enhanced ultrasound
CEUS	contrast-enhanced ultrasound
SPN	solitary pulmonary nodule
VATS	video-assisted thoracoscopy
CCDS	color-coded Doppler sonography
CT	computer tomography
TTP	time to peak
SRI	spectral radiation imaging
AUC	area under the curve
